# Energy-Efficient Algorithm for Sensor Networks with Non-Uniform Maximum Transmission Range

**DOI:** 10.3390/s110606203

**Published:** 2011-06-08

**Authors:** Yimin Yu, Chao Song, Ming Liu, Haigang Gong

**Affiliations:** School of Computer Science and Engineering, University of Electronic Science and Technology of China, Chengdu, 610054, China; E-Mails: allbooks@tom.com (Y.Y.); csmliu@uestc.edu.cn (M.L.); hggong@uestc.edu.cn (H.G.)

**Keywords:** energy hole, load-similar node distribution, energy balance, network lifetime

## Abstract

In wireless sensor networks (WSNs), the energy hole problem is a key factor affecting the network lifetime. In a circular multi-hop sensor network (modeled as concentric coronas), the optimal transmission ranges of all coronas can effectively improve network lifetime. In this paper, we investigate WSNs with non-uniform maximum transmission ranges, where sensor nodes deployed in different regions may differ in their maximum transmission range. Then, we propose an Energy-efficient algorithm for Non-uniform Maximum Transmission range (ENMT), which can search approximate optimal transmission ranges of all coronas in order to prolong network lifetime. Furthermore, the simulation results indicate that ENMT performs better than other algorithms.

## 1. Introduction

Usually, sensor nodes are powered by batteries and once the battery energy is consumed, the nodes cannot work anymore. That is to say, the main constraint of sensor nodes is their finite and low battery energy. Since sensor nodes are often deployed in hostile or harsh environments such as battlefields, deep sea, volcanos, it is often difficult or impossible to recharge or replace the batteries. In order to prolong the network lifetime, the algorithms for wireless sensor networks must be energy efficient.

Most research works for wireless sensor networks often assume that the data collected by sensors are transmitted to one or several sink nodes in some specific location in the WSNs. It was noticed that the sensors closest to the sink tend to deplete their energy budget faster than other sensors [1], which creates an energy hole around the sink. Once an energy hole appears, no more data can be transmitted to that sink. Consequently, a considerate amount of energy is wasted and the network lifetime ends prematurely. Experiments in [2] showed that there is still a great amount of energy left unused after the network lifetime is over for large-scale networks, which can be as much as 90% of total initial energy. Consequently, improving the energy efficiency and prolonging the lifetime of networks is a key problem.

There have been a lot of research works on solving the energy hole problem, most of which assume that sensors nodes have the same capabilities such as radio and initial energy. However, the network is heterogeneous in some applications. For example, sensor nodes deployed in different regions may differ in their maximum transmission range, which we called non-uniform maximum transmission range. In this paper, we analyze the energy hole problem for wireless sensor networks with non-uniform maximum transmission ranges and we propose an energy-efficient algorithm in sensor networks with non-uniform maximum transmission range (ENMT) to solve the energy hole problem. Simulation results show that ENMT consumes the energy efficiently and prolongs the network lifetime in a heterogeneous network.

The rest of the paper is organized as follows. Section 2 reviews the related works. Section 3 introduces the system model and describes the energy hole problem of sensor networks with non-uniform maximum transmission range. Section 4 presents the ENMT algorithm in detail. Simulation results are discussed in Section 5. Section 6 concludes the paper.

## 2. Related Works

Recently, research on wireless sensor networks has indicated that the energy of sensor nodes is consumed unevenly in the network. In [3] Perillo *et al*. discuss two uneven energy consumption problems in sensor networks with uniform node distribution. One assumes that all nodes send data to the sink node directly, so that the nodes that are further away from the sink consume much more energy than the nodes closer to the sink. The other assumes that all nodes transmit their data to the sink node by multi-hop. However, this way the nodes closer to the sink node will deplete their energy quickly because they must relay much more data from other nodes to the sink node.

Li and Mohapatra [4] study the uneven energy consumption problem in a large-scale wirelss sensor network with many-to-one communication. In many-to-one communication networks, all sensor nodes generate data at a constant rate, and data will be sent to the sink node at the same rate by multi-hop. The authors describe the energy hole in a ring model (like corona model), and define the per node traffic load and the per node energy consumption rate (ECR). Obviously, sensor nodes near the sink node have more traffic load. Their analysis proved that sensor nodes in the inner rings consume energy faster than those in the outer rings so that they have shorter lifetimes. The phenomenon of uneven energy consumption is called the energy hole problem, which may result in serious consequences, e.g., early dysfunction of an entire network. The authors present some approaches to solve the energy hole problem, including deployment assistance, traffic compression and aggregation.

Wu and Chen [5] propose a non-uniform node distribution strategy to manage sub-balanced energy depletion. The authors state that if the number of nodes in coronas increases from corona *C_R-1_* to corona *C_1_* in geometric progression with a common ratio *q > 1*, and there are *N_R-1_/(q − 1)* nodes in corona *C_R_*, then the network can achieve sub-balanced energy depletion. Here, *Ci* is the sub-area delimited by the circles of radius *r_i-1_* and *r_i_*, *N_i_* denotes the number of nodes in corona *C_i_*. However, the node distribution strategy can hardly work in the real world, because in most cases the node distribution is random, and hence the node density in local area is uncontrollable.

Olariu and Stojmenovic [1] discuss the relationship between the network lifetime and the width of each corona in a concentric corona model. The authors prove that in order to minimize the total amount of energy spent on routing along a path originating from a sensor in a corona and ending at the sink, all the coronas must have the same width. However, the authors assume that all nodes in corona *C_i_* should forward data in corona *C_i-1_*, and the transmission range in corona *C_i_* is *(r_i_ − r_i-1_)*.

For balancing the energy load among sensors in the network, Jarry *et al*. [6] propose a mixed routing algorithm which allows each sensor node to either send a message to one of its immediate neighbors, or to send it directly to the base station, and the decision is based on a potential function depending on its remaining energy. However, when the network area radius is bigger than the sensor’s maximal transmission range, the proposed algorithm is not applicable. Shiue *et al*. [7] also propose an algorithm to resolve the energy hole problem which uses mobile sensors to heal energy holes. However, the cost of these assistant approaches is high.

## 3. Network Model and Problem Analysis

In this section, we’ll present the system model including the network model and energy model first. Then an improved corona model for sensor networks with non-uniform maximum transmission range is proposed, which introduces the idea of a hierarchy into the traditional corona model.

### 3.1. Network Model

We assume that sensor networks have the following characteristics:

1. All sensor nodes are left unattended after being deployed and remain static.
2. The initial energy of sensor nodes is set to a specific value with no battery recharging or replacement. That is to say, the energy of sensor nodes can’t be refreshed. Once energy is consumed, the nodes doesn’t work anymore.
3. Sensor nodes in different coronas have a different maximum transmission range. The maximum transmission range of the *i*th corona is denoted by *tx_i_*, which is much smaller than *R* (the furthest possible distance from a sensor to its closest sink).
4. Power control is available. Sensor nodes use different radio power to transmit data to different ranges.
5. Sensors send their data at a certain rate. For simplicity, we assume that each sensor node generates and sends *l* bits of data per unit time.
6. Based on the definition of network lifetime in [4], we define the network lifetime in this paper as the duration from the very beginning of the network until the first corona of sensor nodes dies. A corona of sensor nodes in the network is said to be dead when it is unable to forward any data or send its own data.

The notations used in this paper are shown in Table 1.

### 3.2. Energy Model

A typical sensor node is composed of three basic units: sensor unit, processing unit, and wireless radio unit. The energy consumption of the sensor unit and processing unit can be neglected compared to the wireless radio unit. In this paper, we only consider the energy consumption of the wireless radio, which consumes energy for sending and receiving data. We use the same radio model in [4] to analyze the energy hole problem. To transmit a *l*-bit message a distance *d*, the radio expends energy as in Equations (1) and (2).

(1) $$E_{\text{trans}}=(\beta_{1}+\beta_{2}d^{\alpha })l$$

(2) $$E_{\text{rec}}=\beta_{3}l$$

Here, *E_trans_* denotes energy consumption when transmitting data and *E_rec_* denotes energy consumption when receiving data; *l* is the data rate of generating and sending data for sensor node; *α* is 2 or 4, and the term *d^α^* accounts for the path loss. The further the transmission range, the more energy is consumed. According to [4], some typical values for the above parameters in current sensor technologies are as follows:

$$\begin{array}{l} \beta_{1}=45\times 10^{-9}\;\text{J}/\text{bit},\\ \beta_{2}=10\times 10^{-12}\;\text{J}/\text{bit}/{\text{m}}^{2}\;(\text{when}\;\alpha =2),\\ \text{or}\;\beta_{2}=0.001\times 10^{-12}\;\text{J}/\text{bit}/{\text{m}}^{4}\;(\text{when}\;\alpha =4),\\ \beta_{3}=135\times 10^{-9}\;\text{J}/\text{bit}. \end{array}$$

### 3.3. Problem Statement

**Figure 1.**
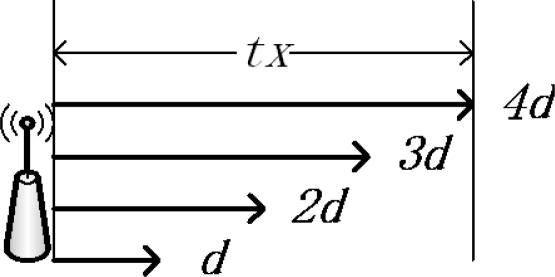
The adjustable transmission range.

Sensors can use different levels of radio power to send their data to different transmission ranges in order to save energy. Each node has a maximum transmission range *tx_i_*, and *i* is the number of the corona in which the node located. For simplicity, we divide the maximum transmission range *tx_i_* into *k_i_* levels (see Figure 1). Then each sensor has *k_i_* levels of transmission range to choose from. The unit length of transmission range is denoted by *d* in Equation (3). As in Figure 1, the node’s transmission range is divided into four levels. The maximum transmission range *tx* is *4d* so that the node can send its data to the distance *d*, *2d*, *3d* and *4d* by controlling its radio power:

(3) $$d={tx}_{i}/k_{i}$$

**Figure 2.**
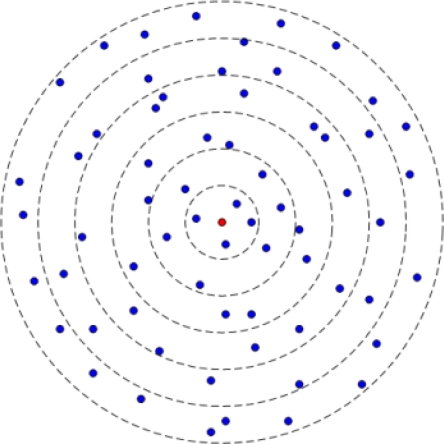
Concentric corona model.

As shown in Figure 2, we partition the whole region with radius *R* into *m* adjacent concentric parts called coronas like in [8]. The width of each corona is *d* and *C_i_* denotes the *i*th corona. The corona *C_i_* includes all nodes whose distances to the sink nodes are between (*i* − 1) *d* and *id.* Therefore:

(4) m=R/d

We assume that all nodes in the same corona have the same transmission range which is called the transmission range of the corona and the nodes in different corona have different transmission ranges. So, when transmission range level of a corona is *j*, the actual transmission range of the sensor satisfies:

(5) $$j\times d=j\times ({tx}_{i}/k_{i})$$

**Figure 3.**
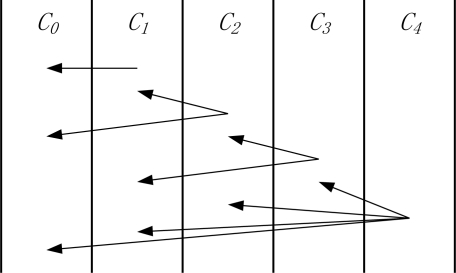
Sensor networks with non-uniform maximum transmission range.

Let *x_i_* denote the transmission range level of the nodes in corona *C_i_* (*x_i_* = 1, 2, …, *k_i_*) and *k_i_* denote the maximum transmission range level for this corona. As shown in Figure 3, *k_1_* = *k_2_* = *k_3_* = 2, and *k_4_* = 4.

Let *N_i_* denote the number of nodes in corona *C_i_*. *S_i_* denotes the set of corona *ID* for the coronas which directly transmit data to the corona *C_i_* and can be expressed by

(6) $$S_{i}=\{k|k-x_{k}=i,\;k=1,2,\ldots ,m\}$$

As shown in Figure 3, taking the corona *C_1_* as an example, the corona *C_2_* with transmission range 1 (*i.e.*, *k* = 2, *x_k_* = 1) can directly transmit data to it. Therefore, in Figure 3, *S_1_* = {2, 3, 4}.

The nodes in each corona not only transmit the data it has generated by itself, but also forward data from the nodes in outer coronas. Let *D_rec i_* denote the data received from outer coronas by the nodes in corona *C_i_* per unit time and it satisfies:

(7) $$D_{\text{rec}i}(\vec{x})=\{\begin{array}{ll} \sum_{j\in S_{i}}(N_{j}L_{j}+D_{\text{rec}\;j}), & if\;\;S_{i}\neq \phi \\ 0, & if\;\;S_{i}=\phi \end{array}$$

Assuming that sensor nodes in corona *C_i_* generate and transmit *L* bits data per unit time, the energy consumption of forwarding data from outer coronas to corona *C_i_* includes the energy consumption for receiving and transmitting. According to the energy equation described in Section 3.2, the total energy consumption of forwarding data generated from other coronas per unit time satisfies:

(8) $$E_{\text{trans}\;i}(\vec{x})=N_{i}L[\beta_{1}+\beta_{2}{\left(x_{i}d\right)}^{\alpha }]\;\;\;\;\;\;\;\;\;\;x_{i}=1,2,\ldots ,k_{i}$$

For corona *C_i_*, the energy used for forwarding data from outer coronas composes of energy for receiving data and sending data. According to Equations (1) and (2), the total energy consumption for corona *C_i_* to forward data generated by outer coronas is:

(9) $$E_{\text{forward}\;i}(\vec{x})=D_{\text{rec}\;i}(\vec{x})[\beta_{1}+\beta_{2}{\left(x_{i}d\right)}^{\alpha }+\beta_{3}]\;\;\;\;\;\;\;\;x_{i}=1,2,\ldots ,k_{i}$$

Let *E_i_* denote the total energy consumption for corona *C_i_* per unit time. *E_i_* includes the energy for transmitting data generated by itself and that for forwarding data from outer coronas and it satisfies:

(10) $$E_{i}(\vec{x})=E_{\text{trans}\;i}(\vec{x})+E_{\text{forward}\;i}(\vec{x})$$

With the Equations (8) and (9), *E_i_* could be described as:

(11) $$E_{i}(\vec{x})=N_{i}L[\beta_{1}+\beta_{2}{\left(x_{i}d\right)}^{\alpha }]+D_{\text{rec}\;i}(\vec{x})[\beta_{1}+\beta_{2}{\left(x_{i}d\right)}^{\alpha }+\beta_{3}]\;\;\;\;x_{i}=1,2,\ldots ,k_{i}$$

Let *T_i_* denote the lifetime of corona *C_i_*. It satisfies:

(12) $$T_{i}(\vec{x})=\frac{ɛN_{i}}{E_{i}(\vec{x})}$$

Substituting *E_i_* with Equation (11), there has:

(13) $$T_{i}(\vec{x})=\frac{ɛN_{i}}{N_{i}L[\beta_{1}+\beta_{2}{\left(x_{i}d\right)}^{\alpha }]+D_{\text{rec}\;i}{\left(\vec{x}\right)}_{i}[\beta_{1}+\beta_{2}{\left(x_{i}d\right)}^{\alpha }+\beta_{3}]}\;\;x_{i}=1,2,\ldots ,k_{i}$$

**Figure 4.**
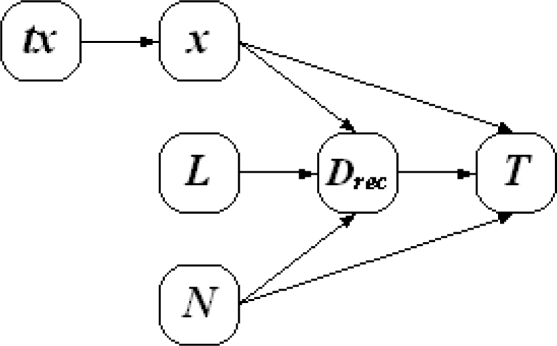
Factors affecting network lifetime.

From Equation (13), we can see there are four factors affecting the network lifetime: the transmission range of the corona *d*, the number of nodes *N_i_*, data generating rate *L* and the amount of data received from outer coronas *D_rec i_*. The relationship of the factors affecting network lifetime is shown in Figure 4. The number of nodes in each corona is determined by the node distribution, and as discussed above the received nodes of each corona is affected by the transmission range list. So after all nodes have been deployed, there is only one factor contributing to the network lifetime, which is the transmission range list. In order to maximize the network lifetime, we need to determine an optimal transmission range list.

## 4. EMNT Design

**Figure 5.**
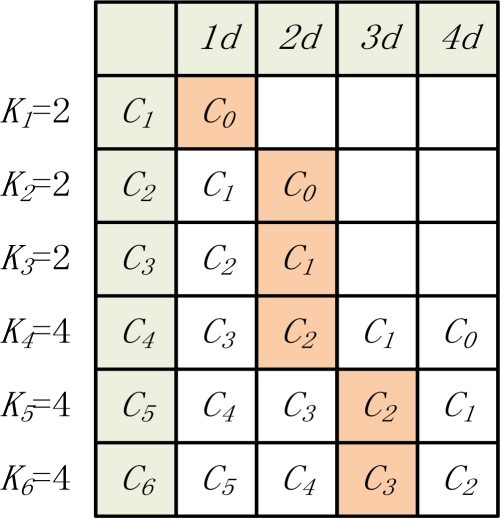
Corona relationship.

As mentioned before, in order to maximize the network lifetime, we need to find an optimal transmission range list for all coronas. Without loss of generality, we discuss it in a sensor network with six coronas. Assuming that the maximum transmission range (*tx*) of the *C_1_*, *C_2_* and *C_3_* is *2d*, and the maximum transmission range (*tx*) of the *C_4_*, *C_5_* and *C_6_* is *4d*, each corona could transmit to other coronas with different transmission ranges. We can build the table of the relationships of all coronas as in Figure 5. For example, the corona *C_1_* is able to transmit data to the sink node by using the transmission range *d*, and its next hop is corona *C_0_* (sink node). For corona *C_5_*, it can choose the transmission ranges *d*, *2d*, *3d*, *4d* to transmit data to the coronas *C_4_*, *C_3_*, *C_2_* and *C_1_*, respectively. Each corona must select a corona as its next hop. In Figure 5, the shaded items are the next hop of each corona. All the shaded items correspond to a transmission range list. For instance, the transmission strategy of each corona in Figure 5 is {*C_0_*, *C_0_*, *C_1_*, *C_2_*, *C_2_*, *C_3_*}. Then the corresponding transmission range list is {1, 2, 2, 2, 3, 3}.

Therefore, in order to prolong the network lifetime, we must find the optimal transmission range list through the corona relationships. We propose ENMT, an energy-efficient algorithm for sensor networks with non-uniform maximum transmission ranges. The goal of ENMT is to find an approximate optimal transmission range list from inner coronas to outer coronas step by step. Because the inner coronas have shorter lifetimes than the outer coronas, ENMT establishes a better transmission range list for inner coronas first. Then the transmission range list of outer coronas is computed to adapt to the transmission range of the inner coronas. Before the network deployment, we can obtain the transmission range list by ENMT algorithm based on deployment information such as radius of the whole monitored area, density of node distribution and data generating rate of each corona. After sensor nodes deployed, the nodes in each corona transmit their data according to the preset transmission rang list. The ENMT algorithm is described as follows:

1. Initialize the table of corona relationship and set each *S_i_* (1 ≤ *i* ≤ *m*) to empty. Add corona *C_0_* into set *S_0_* and set *i* = 0. Here, *S_i_* is set of transmission range lists whose network lifetime approximates to the optimal transmission range list.
2. *i* = *i* + 1. For each corona *C_i_*, try to add its next hop according to the table of corona relationships into the set *S*_*i*-1_ and build some temporary list combined with the lists in *S_i-1_*. Obviously, if corona *C_i_* has *q* coronas as its next hop and the set *S_i-1_* has *p* transmission range lists, there will be *q* × *p* temporary lists. According to these temporary lists, compute the corresponding network lifetime *t* and add it into set *T_i_*.
3. Assuming *T_max_* is the maximum network lifetime in the set of *T_i_*. Add the temporary lists whose network lifetime are between *T_max_* and *T_max_* × (1 − *TIMERANGE*) to *S_i_*. Here, parameter *TIMERANGE* denotes the percentage of *T_max_* which is used to determine the range of temporary lists added to *S_i_*. If the number of selected temporary lists is more than *MAXCOUNT*, then just add *MAXCOUNT* temporary trees whose network lifetime is longer than others to *S_i_*. Here, *MAXCOUNT* denotes the upper limited number of lists in *S_i_*.
4. If *i* is equals to the number of coronas *m,* then select the transmission range lists with the maximum network lifetime in *S_m_* as the final results; if not, go to step (2) for the next loop.

We notice that the computational performance of the algorithm is affected by the parameter of *MAXCOUNT*. For the set *S_m_*, the number of the computed lists is less than (*MAXCOUNT* × *m*). As shown in Figure 5, the *m* in the WSN is 6. Take *C_3_* as an example, *S_2_* = {(*C_0_*, *C_1_*), (*C_0_*, *C_0_*)}. Then we add *C_2_* and *C_1_* into *S_2_* for building *S_3_*, so *S_3_* = {(*C_0_*, *C_1_*, *C_2_*), (*C_0_*, *C_0_*, *C_2_*), (*C_0_*, *C_1_*, *C_1_*), (*C_0_*, *C_0_*, *C_1_*)}. Finally, we can obtain the set *S_6_*, and select the transmission range lists with the maximum network lifetime in *S_6_* as the final results (e.g., {*C_0_*, *C_0_*, *C_1_*, *C_2_*, *C_2_*, *C_3_*}).

## 5. Simulation Results

In order to evaluate the performance of ENMT, we simulated the ENMT algorithm. According to [1], two other algorithms are simulated as the baseline. One is *Max*, in which all sensor nodes transmit using the maximum transmission range *tx* and sensor nodes whose distance to sink node is less than *tx* should send their data to sink node directly. The other is *Same*, in which all senor nodes transmit using the same transmission range that is the minimum transmission range among transmission ranges of the coronas.

In the simulation, the initial energy of each sensor node is 50 J. The level of the maximum transmission range is set to 4 and the density of nodes is 5 nodes/m^2^. In the ENMT algorithm, MAXCOUNT is set to 200. The parameters of energy model such as *β*_1_, *β*_2_, *β*_3_ are set to the values in Section 3.2, and the parameter of α is set to 4.

**Figure 6.**
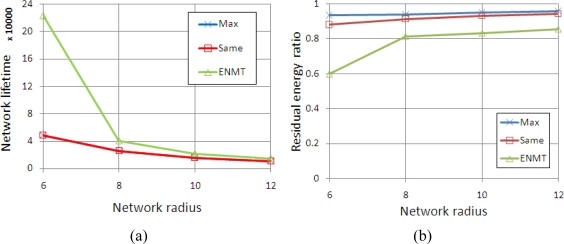
Simulation results (2 and 4). **(a)** Network Lifetime. **(b)** Residual energy ratio.

We conduct simulations in four scenarios corresponding to the networks with 6, 8, 10 and 12 coronas (*m*). The coronas in networks are categorized into two groups and each group contains the successive *m*/2 coronas. The maximum transmission range is set to 2 and 4 from inner group to outer group. Figure 6 shows the network lifetime and residual energy ratio with different algorithms. We can see that the network lifetime with the three algorithms decreases with the growth of network radius. This is because the data traffic is increasing while the radius is increasing, especially for the inner coronas. In Figure 6(b), we note that while the network radius is increasing the lifetime of network is decreasing, but the total initial energy is increasing, so the residual energy ratio is slowly increasing. Obviously, the ENMT algorithm performs better than the other two algorithms both in network lifetime and residual energy ratio.

**Figure 7.**
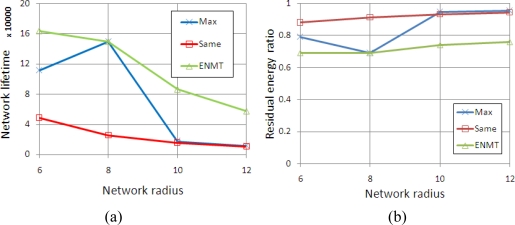
Simulation results (4 and 2). **(a)** Network Lifetime. **(b)** Residual energy ratio.

Different from the simulations in Figure 6, we change the maximum transmission range to 4 and 2 from inner group to outer group. Figure 7 shows the network lifetime and residual energy ratio with different algorithms. Obviously, the ENMT algorithm also performs better than the other two algorithms both in terms of network lifetime and residual energy ratio.

**Figure 8.**
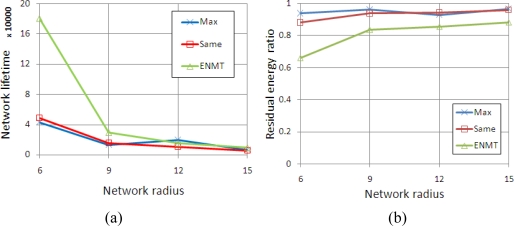
Simulation results (2, 3 and 4). **(a)** Network Lifetime. **(b)** Residual energy ratio.

We conduct simulations in four scenarios corresponding to the networks with 6, 9, 12 and 15 coronas (*m*). The coronas in networks are categorized into three groups and each group contains the successive *m*/3 coronas. The maximum transmission range is set to 2, 3 and 4 from inner group to outer group. Figure 8 shows the network lifetime and residual energy ratio with different algorithms. We notice that the ENMT algorithm outperforms than the other two algorithms both in network lifetime and residual energy ratio.

**Figure 9.**
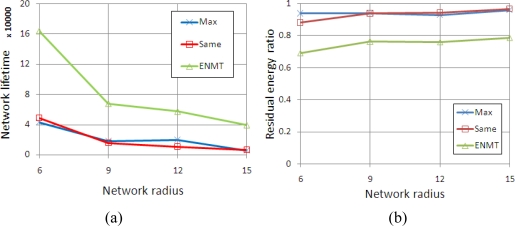
Simulation results (4, 3 and 2). **(a)** Network Lifetime. **(b)** Residual energy ratio.

Different from previous simulations, the maximum transmission range is set to 4, 3 and 2 from inner group to outer group. Figure 9 shows the network lifetime and residual energy ratio with different algorithms. Obviously, the ENMT algorithm also outperforms than the other two algorithms both in network lifetime and residual energy ratio.

## 6. Conclusions

The energy hole problem greatly shortens the lifetime of sensor networks and leads to much residual energy being wasted in networks. In this paper, we have proposed an energy-efficiency algorithm called ENMT to solve the energy hole problem for sensor networks with non-uniform maximum transmission ranges. The goal of ENMT is to find a better transmission range list that can prolong the network lifetime. Simulation results show that ENMT outperforms other algorithms and could effectively prolong the network lifetime.

## Figures and Tables

**Table 1.** The notations used in this paper.

**Notation**	**Physical Meaning**
*C_i_*	The sub-area delimited by the circles of radius *r_i-1_* and *r_i_*
*N_i_*	The number of nodes in corona *C_i_*
*tx_i_*	The maximum transmission range of the *i*th corona
*r_i_*	The radius of the *i*th circle
*l*	Each sensor node generates and sends *l* bits of data per unit time
